# Beginning of ABO-Incompatible Transplants in Pakistan: A Single-Centre Experience

**DOI:** 10.7759/cureus.91831

**Published:** 2025-09-08

**Authors:** Zaeema Ahmad, Andleeb Raja, Nubair Sarwar, Munawal Javid, Naveed Sarwar, Sobia Mukhtar, Mubashar Nazar, Mishal Imran, Anum Mazhar Qureshi, Sara Sarfraz

**Affiliations:** 1 Nephrology, Bahria International Hospital (Safari), Rawalpindi, PAK; 2 Emergency Medicine, Bahria International Hospital (Safari), Rawalpindi, PAK; 3 Urology, Armed Forces Institute of Urology, Rawalpindi, PAK; 4 Urology, Bahria International Hospital (Safari), Rawalpindi, PAK

**Keywords:** abo incompatible renal transplant, challenges in resource limited settings, cost-effectiveness, pakistan, renal transplant, survival rate

## Abstract

Background

The burden of end-stage renal disease (ESRD) is ever-increasing in Pakistan. Among the treatment options available, kidney transplantation remains the most effective treatment for ESRD, offering improved quality of life and survival benefits over dialysis. ABO blood group incompatibility has traditionally been viewed as a barrier to transplantation. However, we have started ABO-incompatible (ABOi) renal transplant in Pakistan since 2023 with good clinical outcomes.

Objectives

This study aims to document the initiation of ABOi renal transplants in Pakistan, while evaluating clinical outcomes, immunological responses, and long-term graft survival rates, with a focus on the development and further improvement of pre-transplant desensitisation regimens.

Methods

A retrospective study was conducted at our centre on ABOi renal transplants from February 2023 to March 2025. A total of 15 ABOi renal transplants were included. The desensitisation protocol developed included immunomodulation with rituximab, followed by low-dose immunosuppression and membrane plasma separation for preformed antibody depletion. Induction therapy was performed using anti-thymocyte globulin (ATG). Immunosuppression consisted of tacrolimus (0.1 mg/kg/day), mycophenolate mofetil or MMF (2000 mg/day), and prednisolone (1 mg/kg/day). All patients were given trimethoprim-sulfamethoxazole 160/800 on alternate days for six months as prophylaxis against Pneumocystis jirovecii pneumonia (PJP), valganciclovir 450 mg per day against cytomegalovirus (CMV) for three months, and fluconazole 50 mg per day for one month as antifungal prophylaxis.

Results

The highest antibody titers pre-transplant were 1/512 (IgG) and 1/256 (IgM). Five sessions of plasmapheresis were done for 13 (86.7%) patients, while two (13.3%) underwent immunoadsorption (IA). The cutoff of 1:4 was established to go ahead with the transplant. Overall graft survival was 86.7%. Three (20.0%) patients developed cellular rejection, which was successfully treated. One (6.7%) patient died with a functioning graft due to secondary complications. The main complications encountered were development of lymphocele, urinary leakage, lower respiratory tract infection, and urinary tract infection.

Conclusion

We conclude that the ABOi renal transplants in Pakistan show favourable results. Further adoption of this method can expand the donor pool for patients with ESRD.

## Introduction

The burden of end-stage renal disease (ESRD) is ever-increasing globally, and more so in developing countries like Pakistan. A study by Mills et al. showed that the age-adjusted prevalence of chronic kidney disease (CKD) was higher in low- and middle-income countries compared to high-income countries [1]. Pakistan is seeing an alarming increase in the number of patients with renal disease. It is estimated that 25,000 persons suffer from renal failure every year, out of which only 10 percent succeed in receiving dialysis, and only 2.3 percent are lucky to receive transplantation [2]. Among the treatment options available, kidney transplant remains the most effective treatment for ESRD, offering improved quality of life and survival benefits over dialysis, as stated in a systematic review by Tonelli et al. [3]. However, the shortage of compatible organ donors poses a significant challenge in the face of an expanding pool of recipients. ABO blood group incompatibility has traditionally been viewed as a barrier to transplantation, leading to missed opportunities for patients who could benefit from a transplant from an incompatible donor.

Recent advances in immunosuppressive therapy and desensitisation protocols have made ABO-incompatible (ABOi) kidney transplantation a viable option. This has led to a wider utilisation of ABOi kidney transplantations, first in Japan from the late 1980s, in the US from the mid-1990s, and in Europe from the early 2000s [4]. Regionally, Nepal and Bangladesh started ABOi kidney transplantation in 2017 and 2018, respectively [5,6]. In Pakistan, our centre was the first to initiate ABOi renal transplantation in 2023.

This case series aims to document the experiences and outcomes of ABOi kidney transplantation performed at Bahria International Hospital (Safari), Rawalpindi, Pakistan over two years. It seeks to provide valuable insights into the feasibility of ABOi transplantation in Pakistan in terms of the development of local guidelines for pre-transplant desensitisation, clinical outcomes, post-transplant complications, and cost-effectiveness, thereby contributing to the growing body of literature on this important topic.

## Materials and methods

Statistical analysis

This retrospective study was conducted at Safari Hospital on ABOi kidney transplants from February 2023 to March 2025. It included 15 ABOi kidney transplant cases from a single centre. Data were gathered from the hospital database and analysed using Microsoft Excel (Microsoft Corp., Redmond, WA, US). Continuous variables (e.g., age, serum creatinine) were presented as mean and range. Categorical variables (e.g., donor recipient age and gender, blood group combinations, human leucocyte antigen (HLA) matching, and complications) were expressed as frequencies (%). No formal statistical hypothesis tests were performed due to the small sample size. The results are presented to describe the patient cohort and observed outcomes.

Pre-transplant screening

For all donors and recipients, pre-transplant screening included a complete blood count (CBC), serum electrolytes (S/E), liver function tests (LFTs), renal function tests (RFTs), coagulation profile, chest X-ray (CXR), electrocardiography (ECG), and echocardiography. Infection screening, including tuberculosis (TB) via QuantiFERON-GOLD (VIDAS® TB-IGRA, bioMérieux SA, France), Hepatitis B surface antigen, Anti-Hepatitis C Virus (HCV), human immunodeficiency virus (HIV) serology, syphilis serology, cytomegalovirus (CMV) serology, and Epstein-Barr virus (EBV) serology, was done for all donors and recipients. HLA typing of donors and recipients was done to assess the immunological risk. Complement-dependent cytotoxicity (CDC) crossmatch, flow cross-match, Luminex Panel Reactive Antibody (PRA) screen were done to assess donor-specific antibodies (DSAs).

Renal donors were evaluated for 24-hour creatinine clearance. Contrast-enhanced CT (CECT) renal angiograms were done to assess the renal vasculature, and renal diethylenetriamine pentaacetic acid (DTPA) scans were performed for split renal function and estimated glomerular filtration rate (eGFR).

Desensitisation protocol

**Figure 1:**
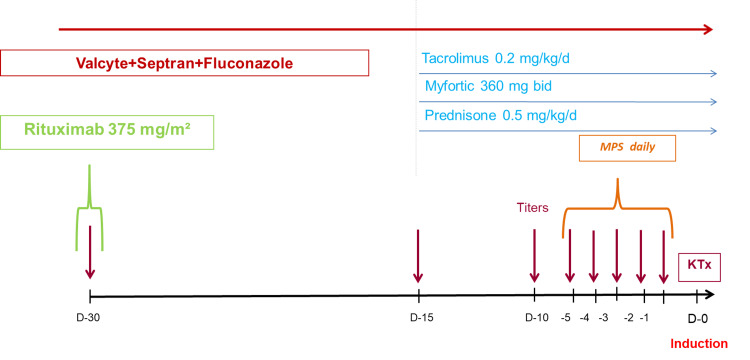
Timeline of the renal transplant desensitisation protocol, outlining immunosuppressive therapy, antibody titers, and induction D: day; MPS: membrane plasmapheresis; KTx: kidney transplant; Valcyte: Valganciclovir; Septran: Trimethoprim/Sulfamethoxazole; Myfortic: Mycophenolate Sodium.

Desensitisation was done starting with rituximab, followed by immunosuppression with tacrolimus and mycophenolate mofetil (MMF), and apheresis immediately before transplant. The desenstisation protocol is outlined with time stamps in Figure 1.

Rituximab

All recipients were given rituximab (anti-CD-20 monoclonal antibody) four weeks before the transplant. The dose was 500 mg diluted in 500 mL of normal saline at the rate of 50 mL/hr, administered over 10 hours under observation with cardiac monitoring. Pre-medication was done with paracetamol 1 g, phenylephrine 22 mg and hydrocortisone 100 mg before the rituximab infusion. Post-rituximab recipients were started on low-dose immunosuppression with tacrolimus and MMF.

Apheresis

Pre-formed antibody depletion was done by three different methods. Traditional plasmapheresis via centrifugation was used for two (13.3%) patients. For 13 (86.7%) recipients, plasma exchange (PEX) was done using a membrane plasma separator (MPS) on a standard hemodialysis machine. Out of these, two (13.3%) patients received them in tandem sessions with consecutive hemodialysis, membrane plasma separation, and immunoadsorption (HD+MPS+IA) and one (6.7%) patient underwent a combined session of MPS and IA, followed by three MPS sessions. Glycosorb columns were used for IA.

Induction

Antithymocyte globulin (ATG) with methyl-prednisolone was given on day 0 till day two of the transplant in divided doses.

Immunosuppression

Tacrolimus (0.1 mg/kg/day), MMF (2000 mg/day), and prednisolone (1 mg/kg/day) were maintained post-transplant.

Isoagglutinin titers

All recipients were tested for pre-transplant antibody titers using gel cards, one month before transplant. Repeat titers were done on the day of the transplant after the last session of PEX. The highest antibody titers observed pre-transplant were 1/512 (IgG) and 1/256 (IgM). The cutoff of 1:4 was established to go ahead with the transplant.

Follow up

All patients remained on triple immunosuppression post-transplantation. Target tacrolimus trough was 8 to 10 ng/ml for the first three months, reduced to 6 to 8 ng/ml in the next three months and maintained on 4 to 6 ng/ml thereafter. MMF was continued at 2000 mg/day. Prednisolone was gradually tapered to a maintenance dose of 5 mg/day.

All patients were given trimethoprim-sulfamethoxazole 160/800 on alternate days for six months as prophylaxis against Pneumocystis jirovecii pneumonia (PJP), valganciclovir 450 mg per day against CMV for three months and fluconazole 50 mg per day for 1.5 months as antifungal prophylaxis.

One patient was given pentamidine for PJP prophylaxis as he was allergic to Septran (trimethoprim-sulfamethoxazole).

Post-transplant monitoring

Patients were followed in out patient department (OPD) on the second day of discharge, then weekly for two months, then every six weeks thereafter, with baseline investigations, CBC, C-reactive protein qualitative (CRP-Q), RFTs, blood sugar random (BSR), alanine aminotransferase (ALT), urine complete examination, and serum tacrolimus trough levels. Antibody titers and renal biopsies were not made a part of routine post-transplant monitoring. The first six patients underwent biopsies as per protocol. Later on, they were only done in case of clinical suspicion supported by a fall in urine output, a rise in creatinine, and raised resistive indices of renal arteries on Doppler ultrasound.

## Results

**Figure 2:**
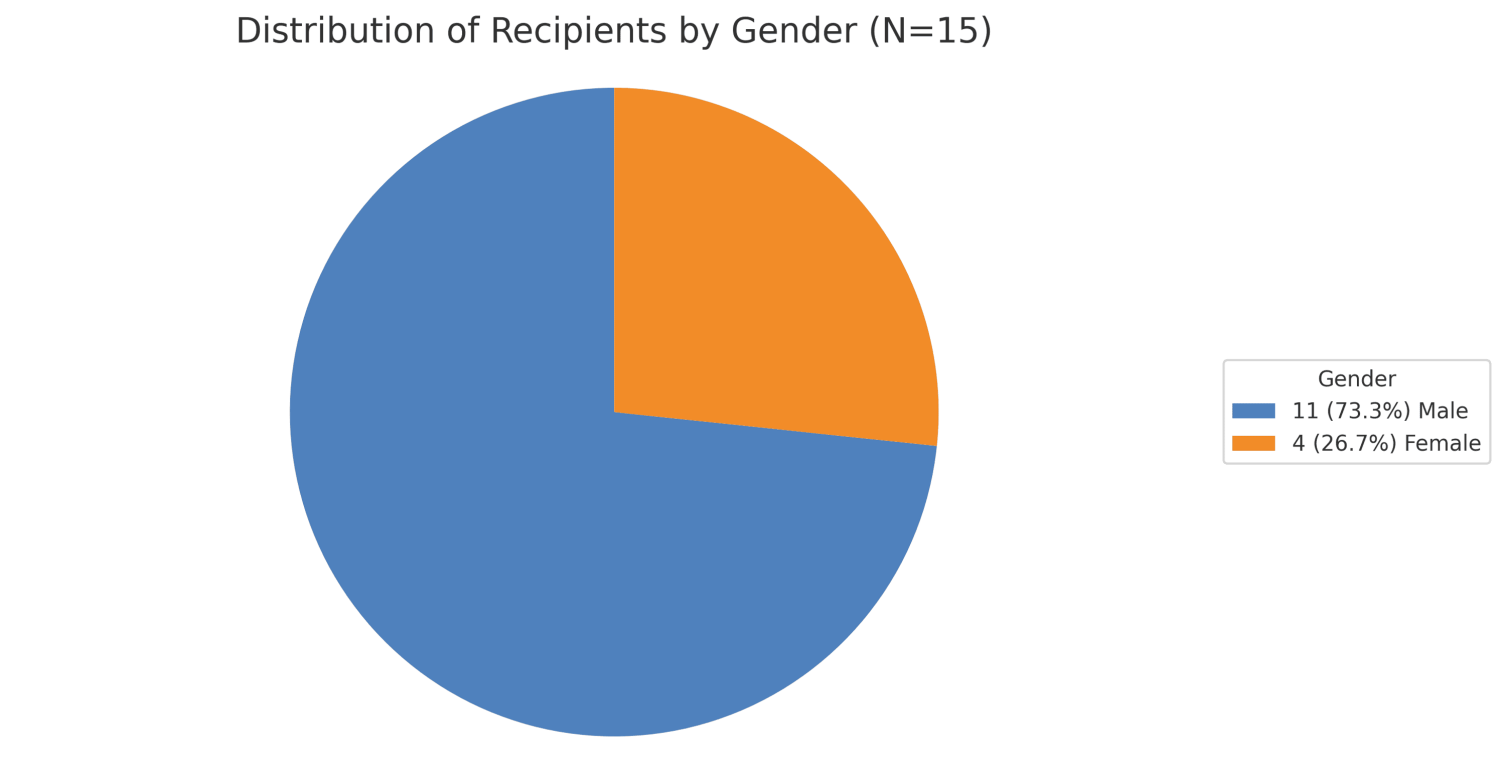
Distribution of recipients by gender

A total of 15 ABOi transplants performed at our centre were studied retrospectively. Eleven (73.3%) of these were male recipients, and four (26.7%) were female recipients. The mean age of the recipient group was between 30 and 40 years. Out of the 15 donors, three (20%) and 12 (80%) were male and female donors, respectively. The mean age of the donors ranged between 35 and 45 years. The pie chart in Figure 2 depicts the gender distribution among the recipients. 

**Figure 3:**
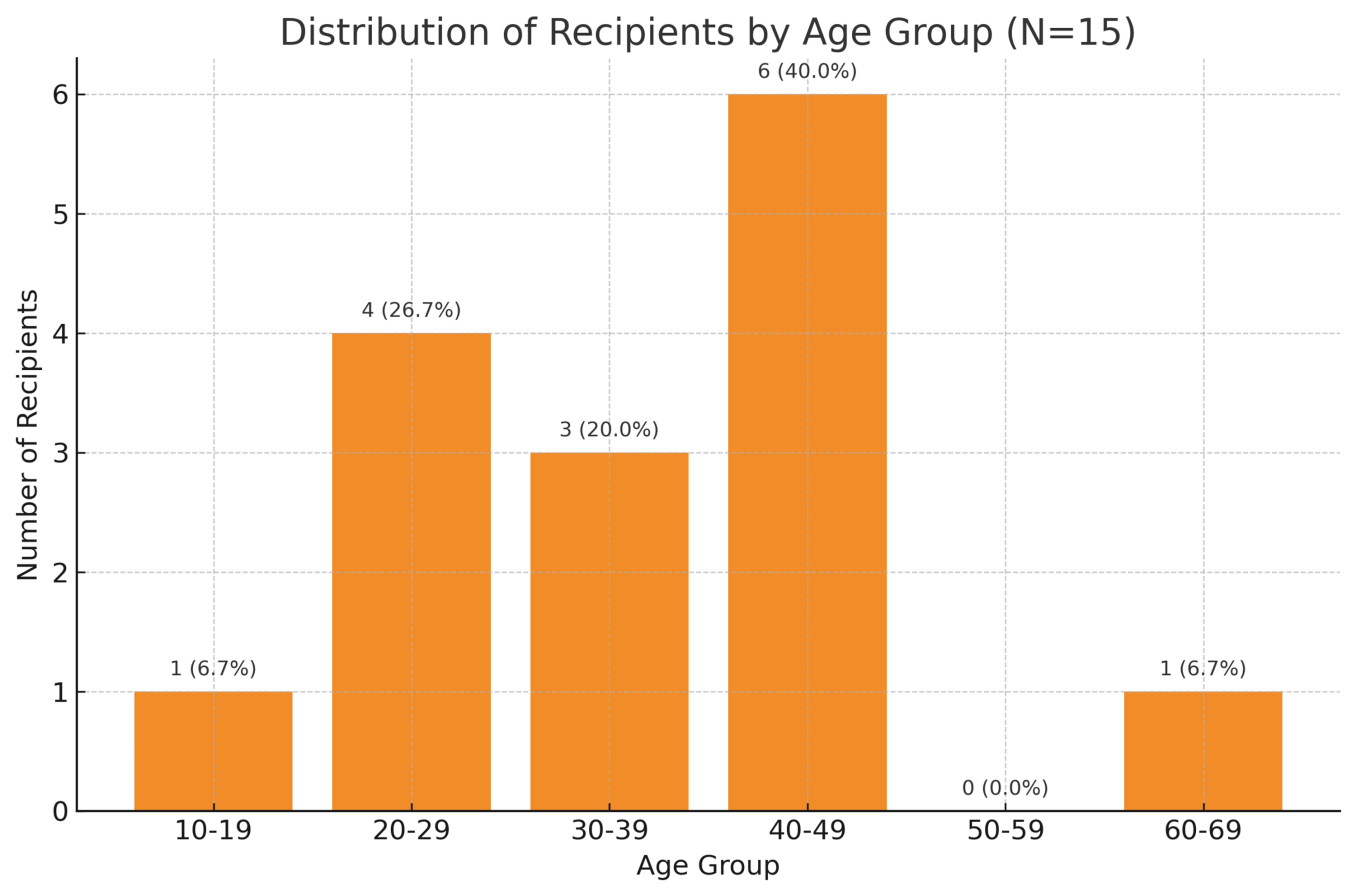
Distribution of the recipients by age group

Figure 3 shows the age distribution of the recipients.

**Figure 4:**
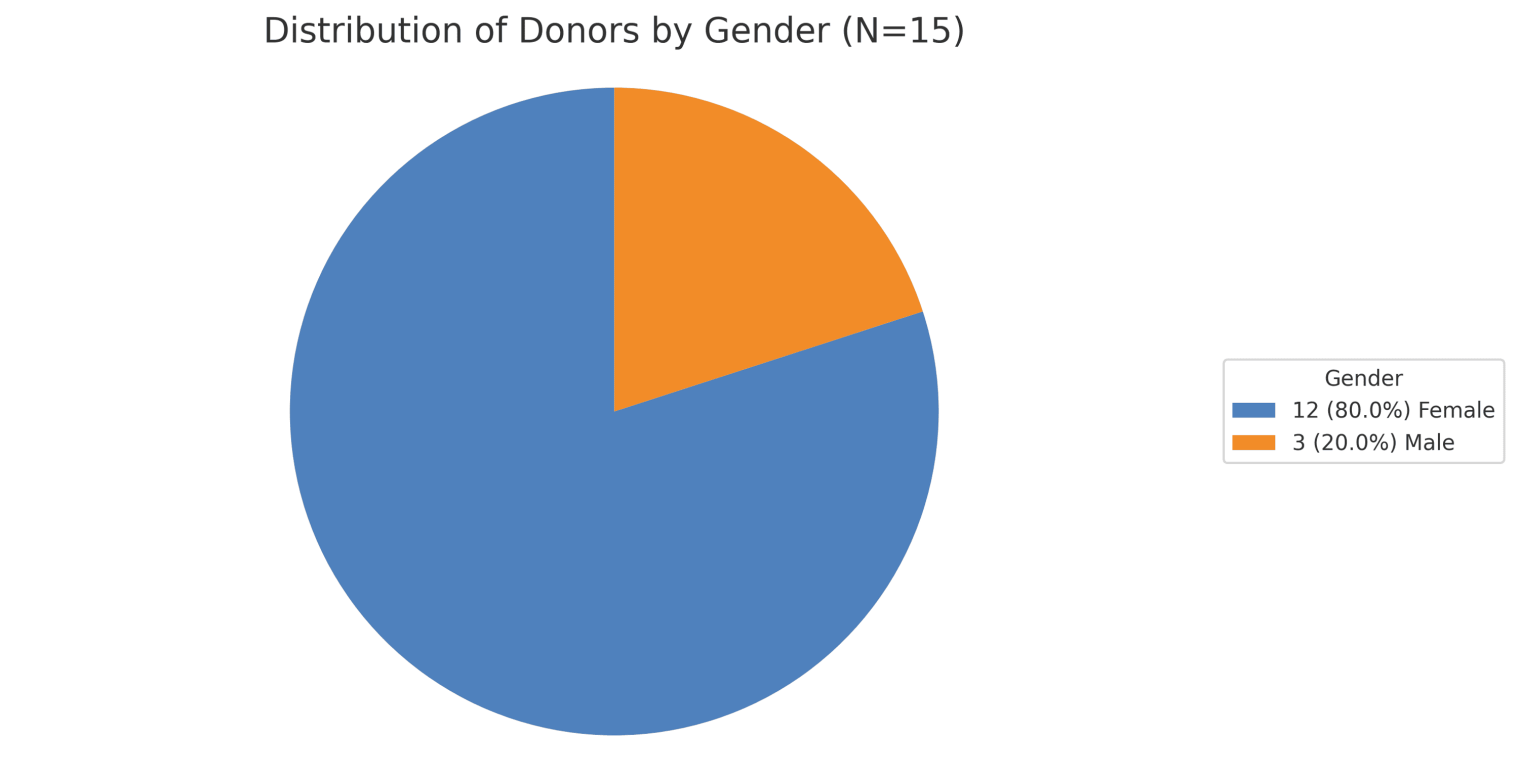
Distribution of donors by gender

Figure 4 illustrates the gender distribution among the donors.

**Figure 5:**
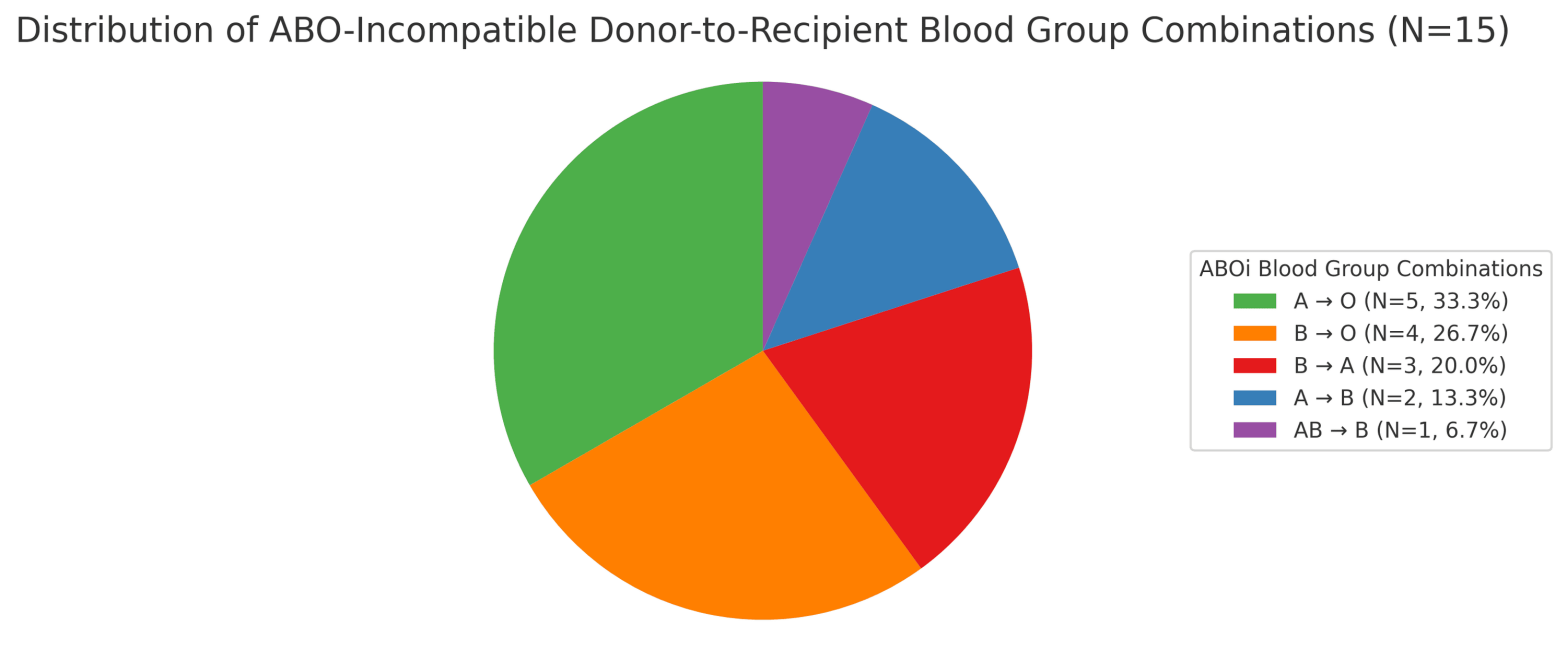
Distribution of blood group combinations

The most common incompatibility, in ABOi donor-to-recipient blood group combinations, was A → O in five cases (33.3%). This means the donor had blood group A and the recipient had group O. The second most frequent was B → O in four cases (26.7%), donor was B, recipient was O. Next was B → A seen in three cases (20.0%), and A → B was observed in two cases (13.3%). The least common was AB → B noted in one case (6.7%). This is illustrated in Figure 5.

In 15 ABOi renal transplants, a significant number of recipients had blood group O. While O is a universal donor, it can only receive from O, which increases the likelihood of requiring ABOi transplants.

Blood group subtyping was performed in six (40%) patients to identify A2 donors; all were found to be A1 subtype. Our study showed that outcomes were not affected by A1 subtype, so further subtyping was not carried out for the remaining donors.

**Figure 6:**
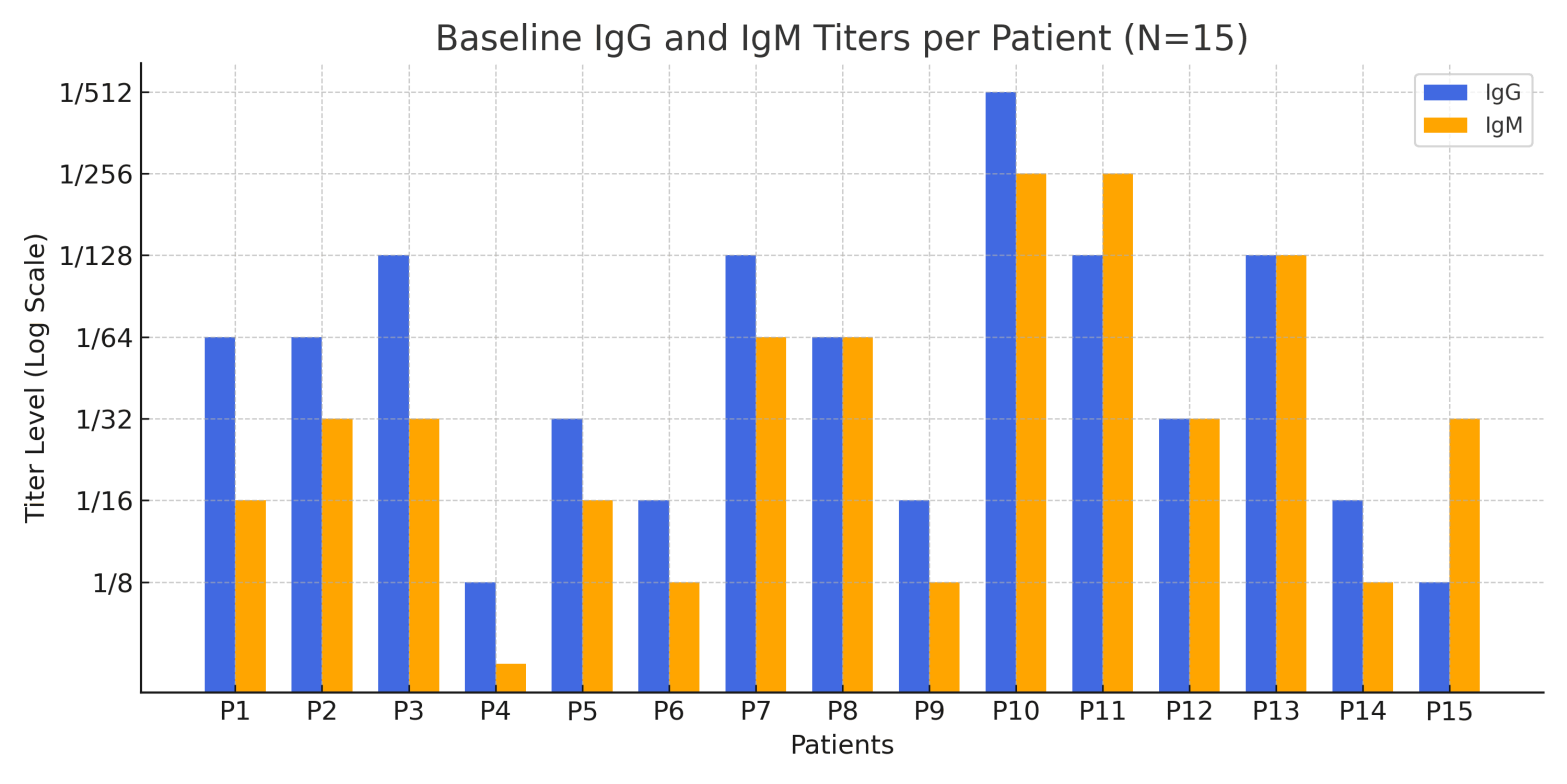
Baseline IgG and IgM titers per patient, showing individual variability in antibody levels on a log scale P: Patient

Pre-transplant baseline antibody titers ranged from 1/8 to 1/512, with a median IgG titers of 1/64 and median IgM titers of 1/32. Overall, IgG titers were higher than IgM as shown in Figure 6.

**Figure 7:**
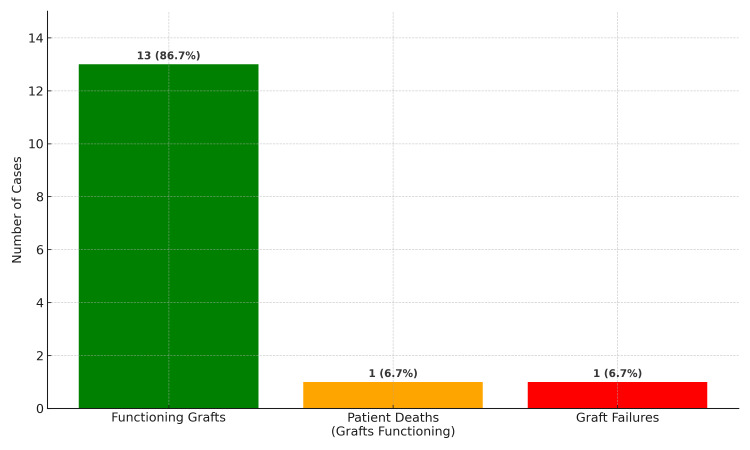
Post-transplant graft outcomes

The graft survival rate was 86.7%, with the longest follow-up being 24 months, depicted in Figure 7.

The mean serum creatinine level was 1.1 mg/dl.

**Figure 8:**
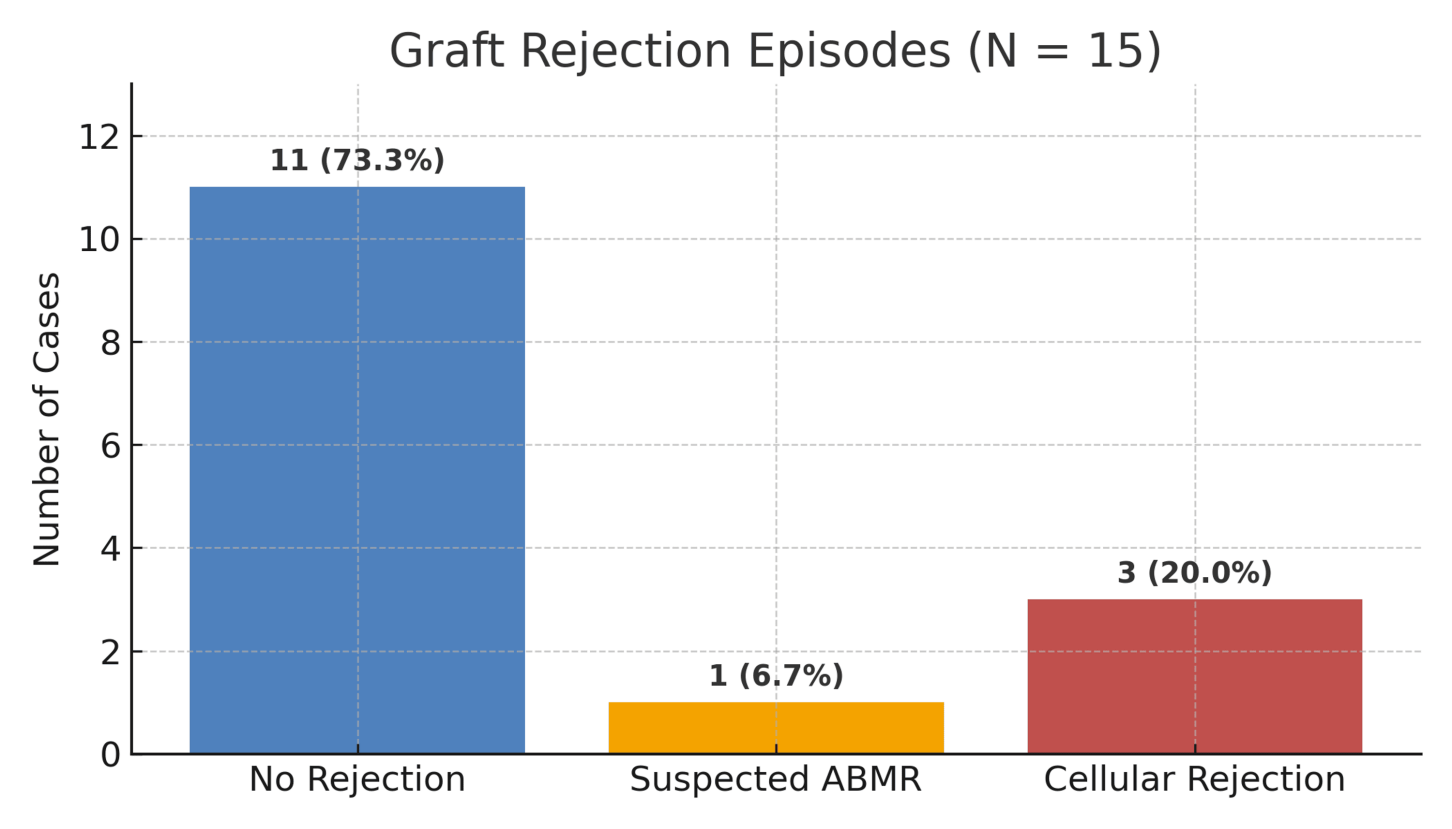
Number of graft rejections and their possible causes ABMR: antibody mediated rejection

Of the 15 patients, four (26.7%) had two haplotype matches with their donors, two (13.3%) had one haplotype match, and the rest nine (60.0%) had less than 50% HLA matching. Despite ABOi, no episodes of hyperacute rejection occurred. However, in four (26.7%) patients, clinical suspicion of acute cellular rejection arose, although this was not confirmed by renal biopsy. Three (20.0%) patients presented with clinical signs of anuria, and their antibody titers were negative. Both responded to a single session of plasmapheresis, which restored urine output within 12 hours. They were concluded to have cellular rejection. One (6.7%) patient experienced suspected acute-antibody mediated rejection that led to death within two weeks, before biopsy confirmation. Figure 8 demonstrates the details of graft rejection.

Another patient died with a functioning graft one month post-transplant due to pulmonary embolism secondary to deep venous thrombosis following a long flight.

Post-operative protocol biopsies were performed in the first six (40.0%) patients on day 30 post-surgery. Only two (13.3%) biopsies showed positive CD4 deposition, while one (6.7%) demonstrated IgA nephropathy. These protocols were not continued in subsequent patients.

Post-transplant antibody titers were checked in four (26.7%) patients. No clinical significance was observed, as rejection was suspected despite negative titers. Therefore, routine titers were not performed unless justified by clinical circumstances.

**Figure 9:**
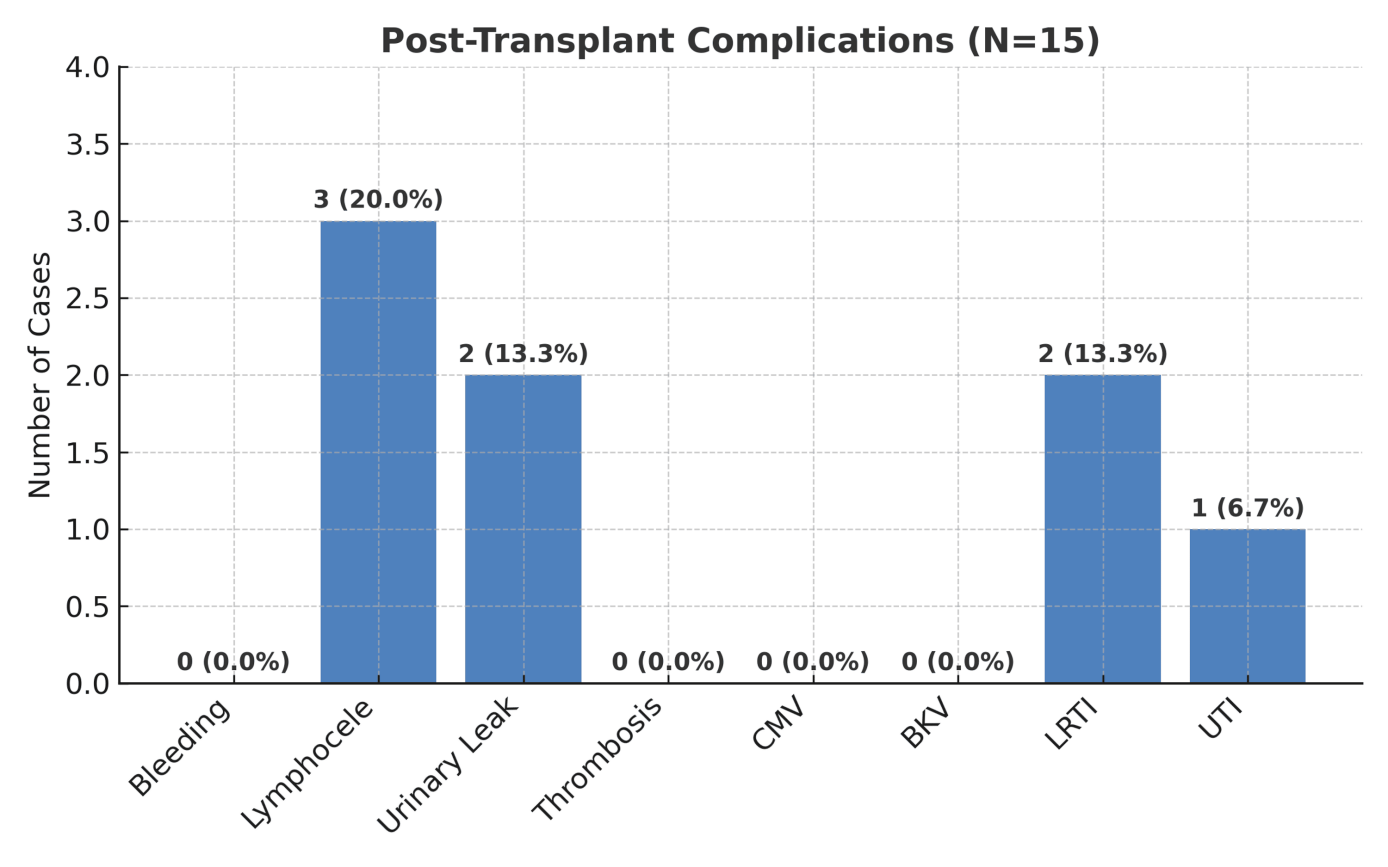
Post-transplant complications CMV: cytomegalovirus; BKV: BK virus; LRTI: lower respiratory tract infection; UTI: urinary tract infection.

Regarding complications, as seen in Figure 9, the most common was lymphocele development in three (20%) patients, which resolved over several months without any intervention.

Two (13.3%) patients experienced urinary leakage requiring surgical intervention, two (13.3%) developed lower respiratory tract infections, and one (6.7%) was treated for a urinary tract infection.

Fourteen (93.3%) patients had normal baseline serum creatinine levels ranging from 0.6 to 0.9 mg/dl at discharge and during follow-up.

## Discussion

Given the disease burden in Pakistan and the improvement in quality of life that transplants provide alongside the region's economic status, renal transplantation offers more benefits than dialysis for patients with CKD. Traditionally, ABO incompatibility was a straightforward barrier to transplantation. The first breakthrough came from Japan in 1987 when the first successful renal transplant was performed using splenectomy as a part of the pre-transplant desensitisation. As reported by Takahashi et al., to meet the strict demands of living donor transplantation, splenectomy is performed at nearly all centers [7]. This approach broadened with the use of rituximab, which eliminated the need for splenectomy. Patients who received the rituximab-containing preconditioning regimen had far lower incidences of acute antibody-mediated rejection and acute cellular rejection, and the regimen did not increase the incidence of CMV infection [8]. However, it is crucial in the management of ABOi kidney transplant to be aware of the risks associated with the high degree of immunosuppression [9]. A meta-analysis showed that one-year uncensored graft survival of patients who were ABOi was 96% versus 98% in ABO-compatible controls [10]. Today, ABOi kidney transplant accounts for approximately 30% of all living donor kidney transplantations performed in Japan [11]. In another study conducted in France, the overall early and long-term outcomes were similar between the groups in terms of patient survival (100%) and graft survival (91% in the ABOi and 99% in the ABO-compatible group), and renal function, within the mean follow-up period of 24 months [12]. The outcome of our center's ABOi renal transplant is similar to that observed regionally and globally. Our overall graft survival was 86.7% within the first two years post-transplant. These results are attributed to the development of a desensitisation protocol tailored to our available resources. From establishing laboratories for antibody titer estimation according to international standards to employing different apheresis methods, it took two years of planning and groundwork to execute the first ABOi kidney transplant.

Hypogammaglobulinemia secondary to immunosuppression is highly prevalent after solid organ transplantation, and intravenous immunoglobulin (IVIG) has been reported to reduce the risks of infections in various settings [13]. However, none of our patients received IVIG, yet there were no cases of BK virus, CMV, Pneumocystis jiroveci, varicella zoster, or fungal infections. The most selective apheresis technique is ABO-specific IA, in which the separated plasma passes through an antigen-specific absorber consisting of blood group A or B antigens immobilised on a sepharose matrix, and then it is returned to the patient [9]. Only two of our patients underwent IA, but this was not feasible for others due to the cost of the column and its single-use nature. Two patients underwent plasmapheresis using the traditional centrifugation technique, while the remaining 12 underwent MPS, which was later established as a mode of plasmapheresis, also performed for the first time in Pakistan. 

The advantages of the primary MPS include its simplicity for use with blood pumps and no observed white blood cell or platelet loss, compared with centrifugation [14]. Fresh frozen plasma was used as a replacement fluid, and no complications were observed during these procedures. Interestingly, most cases of mortality in ABOi kidney transplant developed within six months post-transplantation, and the most common cause of death was infection [15]. Infections, being the most feared complication of this pathway, were not an insurmountable issue. Post-transplant, two patients developed lower respiratory tract infections, and one developed a urinary tract infection. These responded well to empirical antibiotics and resolved without complications. Regarding surgical complications, lymphocele formation was observed in three patients. As documented by a cohort study, ABOi kidney transplant has as good long-term results as ABO-compatible kidney transplant in terms of patient survival, graft survival, and complications, with the exception of increased lymphocele formation [16]. These patients were closely monitored, and the lymphocele resolved without active surgical intervention. However, two patients experienced urinary leakage from the ureteric anastomosis, which was managed surgically.

In a developing country like ours, cost remains a limiting factor compared to ABO-compatible renal transplantation. The average cost of an ABOi transplant in the United States of America is $65,080 compared with $32,039 for an ABO-compatible transplant [5]. In terms of regional cost analysis, a study in Bangladesh reported that the cost of an ABO-compatible kidney transplant was around $3,000, and was approximately $1,900 at public hospitals and $9,900 at private hospitals [6]. In comparison, at our center, desensitisation currently costs approximately $1200. This is partly attributed to the use of MPS as the preferred method of apheresis and using fresh frozen plasma as replacement fluid. Further subsidising the cost of pre-transplant desensitisation could facilitate the wider adoption of this route, increasing the donor pool for many patients awaiting hope. In a local report published by Manzoor et al., the reason behind the development of the international organ trade was stated to be the short supply of indigenous organs [17]. Illegal transplantation is a major problem faced by low-income countries like Pakistan. Facilitating the growth of ABOi transplantation can also curb such practices in the region.

Our study was mainly retrospective and observational, and limited to one center. The small sample size limits the generalisability of the results. The follow-up period was of two years, restricting the assessment of long-term survival and complications. Finally, resource constraints had an impact on development of desensitisation protocols and post-transplant monitoring. This could have potentially influenced the outcomes.

## Conclusions

ABOi renal transplantation is both feasible and effective, even within resource-limited settings. Our experience with 15 patients demonstrated promising outcomes, characterised by low rates of rejection and complications. A standardised desensitisation protocol and structured post-transplant follow-up underpinned the success of the programme. While challenges such as cost constraints, logistical hurdles, and elevated baseline antibody titers persist, they remain manageable with appropriate planning. These findings support the viability of ABOi transplant programmes as a means to broaden access and reduce wait times in countries like Pakistan.
